# The effect of maternal voice and non-nutritional sucking on repeated procedural pain of heel prick in neonates: a quasi-experimental study

**DOI:** 10.1186/s12887-024-04738-7

**Published:** 2024-04-16

**Authors:** Yushuang Chen, Leshan Zhou, Yanjuan Tan

**Affiliations:** 1 Tongji Hospital of Tongji Medical College of Huazhong University of Science and Technology, Wuhan, China; 2 Xiangya Nursing School, Yuelu District, Central South University, No 172, Tongzipo Road, Changsha City, 410013 China; 3 Xiangya Thrid Hospital, Central South University, Changsha, China

**Keywords:** Neonates, Pain relief, Heel lance

## Abstract

**Background:**

Neonates in the neonatal intensive care unit undergo frequent painful procedures. It is essential to reduce pain using safe and feasible methods.

**Purpose:**

To evaluate the effects of non-nutritional sucking, mother’s voice, or non-nutritional sucking combined with mother’s voice on repeated procedural pain in hospitalized neonates.

**Methods:**

A quasi-experimental study was conducted in which 141 neonates were selected in a hospital in Changsha, China. Newborns were divided into four groups: non-nutritional sucking (NNS) (*n* = 35), maternal voice (MV) (*n* = 35), NNS + MV (*n* = 34), and control (*n* = 37) groups. The Preterm Infant Pain Profile-Revised Scale (PIPP-R) was used to assess pain.

**Results:**

During the heel prick, the heart rate value and blood oxygen saturation were significantly different between the groups (*P* < 0.05). Both non-nutritional sucking and maternal voice significantly reduced PIPP-R pain scores of hospitalized newborns (*P* < 0.05). The pain-relief effect was more robust in the combined group than in other groups.

**Conclusions:**

This study showed that both non-nutritional sucking and the mother’s voice alleviated repeated procedural pain in neonates. Therefore, these interventions can be used as alternatives to reduce repeated procedural pain.

## Introduction

Neonatal pain has become an issue that needs to be appropriately addressed, with increasing evidence showing newborns experience frequent and repeated procedural pain in the neonatal intensive care unit (NICU) [1, 2]. When receiving regular treatment, newborns in the NICU undergo many painful procedures, including mechanical ventilation, peripheral venipuncture, heel lancing, etc [3]. Studies showed that newborns experience about 7–17 painful procedures in the first two weeks after birth, and it is established that noise and pain of the NICU have harmful effects and affect infant neurodevelopment [4, 5].

It is estimated that newborns were exposed to extensive and diverse invasive procedures during hospitalization in China; however, most Chinese nurses are unaware of the importance of pain management while implementing potentially painful routine care [6]. Heel lancing is the most frequently used routine painful procedure for newborns. Researchers found that most nurses’ knowledge and attitude concerning pain management are still poor in China, and very few interventions are performed in the clinical setting [7, 8]. Grunau reported that repeated procedural pain in the NICU might contribute to critical long-term effects concerning brain development, neurodevelopment, and later pain sensitivity in newborns [9]. Some studies have demonstrated that the short-term effect of repeated pain exposure on the pain responses of newborns includes continuous crying, blunted cortisol reactivity, changes in heart rate and oxygen saturation, and sleep pattern disturbance [10, 11]. In addition, clinical and animal experiments revealed that untreated repeated pain may result in continuous impairments in adulthood, such as neurodevelopmental disabilities or behavioral changes, because of the alternation of pain conduction pathways in the brain [12]. Existing evidence on the short-term and long-term outcomes of pain has drawn attention to the impact of repeated procedural pain.

Although pharmacologic treatments are effective pain treatments for procedural pain, further studies are needed to prevent associated risks in the most vulnerable neonates [13]. The long-term use of analgesics for neonates can lead to impaired respiratory and cardiovascular functions, followed by drug dependence and withdrawal symptoms [14]. Therefore, non-pharmacologic analgesic interventions are recommended by some national guidelines [15, 16]. Despite the fact that sweet solution, kangaroo care, and maternal feeding are widely acknowledged as effective methods to alleviate repeated procedural pain among neonates, minimal utilization of these strategies was found nationwide [16, 17]. Furthermore, the long-term effects of repeated oral sucrose solutions need further study, and the sweet solution is not suitable for newborns with hyperglycemia. The use of both kangaroo care and maternal feeding is limited to the maternal and infant wards in China, and very few hospitals can provide the opportunity for these neonatal care strategies [18]. Maternal presence is considered an important factor for neonatal pain relief by researchers [19, 20].

To address this issue, researchers are investigating pain management interventions that are more applicable in the NICU. In recent years, the application of music therapy for newborns in the NICU has attracted more and more attention because of its clinical effects. Playing the maternal voice to neonates is a noninvasive and feasible intervention that has been identified as a possible resource for reducing pain-related stress [21]. However, there is some doubt about the effectiveness of this intervention on neonatal pain. Many studies indicated that playing a recording of the maternal voice alone is not sufficient to diminish pain in preterm babies undergoing heel lancing [22, 23]. However, prior research showed that neonates can respond to auditory stimuli after 28 weeks of gestation, and the mother’s voice represented a unique sound stimulus for the fetus [24]. Unlike in the womb, neonates are usually surrounded by many noise disturbances in the NICU and are deprived of appropriate stimuli. The maternal voice is a beneficial auditory stimulus for newborns that provides developmental stimulation during a critical period of growth and promotes parental bonding [21]. Further studies evaluating the effectiveness of the maternal voice for pain relief and desensitization to repeated heel lancing are needed before any conclusions can be drawn.

The use of non-nutritive sucking (NNS) with the aim of stabilizing newborns has been established for painful procedures. Previous studies showed that NNS and music can relieve pain and affect a neonate’s physical stability, although further studies are needed to confirm this [25, 26]. It is unclear if NNS or maternal voice stimulation alone or a combination of the two interventions can effectively relieve the pain caused by repeated heel lancing. NNS or maternal voice may have a certain soothing effect at a single time. Whether the effect of repeated intervention continues to be effective or weakened, the persistence of the effect of repeated intervention needs to be further discussed. Therefore, we aimed to explore the effects of NNS and maternal voice on repeated procedural pain in the NICU.

## Methods

### Participants and setting

**Fig. 1:**
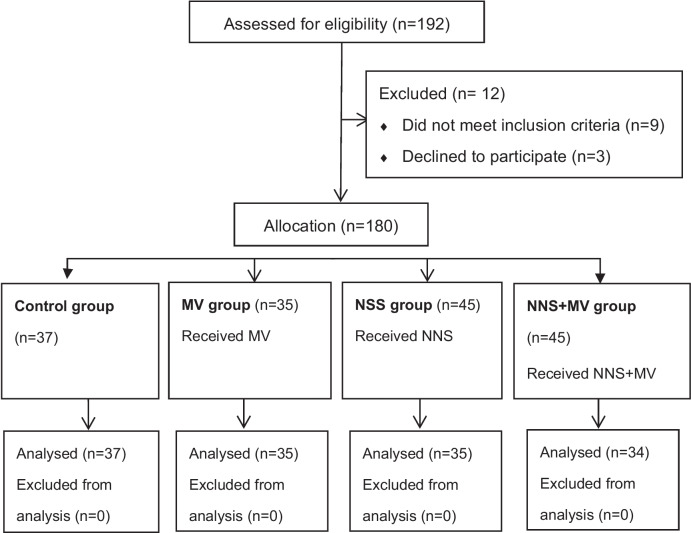
Shows the experimental flow diagram

Neonates were recruited by convenience sampling from a tertiary hospital NICU of Xiangya Medical School between December 2018 and May 2019 in Changsha, China. For this quasi-experimental study, participants were selected based on a sequence of admissions. Neonates received NNS, MV, NNS + MV, and no intervention in a random order each time after a heel prick procedure for a weekly selection. We randomly choose the order of intervention, such as the first week of NNS and the second week of sound intervention. Neonates were included if they met all of these inclusion criteria: (1) gestational age ≥ 28 weeks; birth weight > 1500 g; (2) Apgar score ≥ 8; (3) normal hearing; (4) quiet and pain-free before the procedure and did not receive analgesia, sedation, or oxygen inhalation; (5) estimated to have three or more heel blood collections during hospitalization. Neonates were excluded if they had any of the following criteria: (1) serious infections, shock, fever, or other critical diseases; (2) serious life-threatening complications; (3) crying for unknown reasons before puncture; (4) unsuccessful heel blood collection. The minimum sample size of repeated measurement data was calculated by Power Analysis and Sample Size (PASS) 15 software, and the test efficiency was 0.8, and α = 0.05. Considering a possible drop-out rate of 20%, a total of 32 newborns for each group was recommended by statistics professionals for this study. The allocation procedure is described in Fig. 1.

### Experimental procedures

According to routine health procedures, newborns were placed in the supine position in incubators. Before the heel-prick test, standard daily care such as diaper changes, milk feeding, and bathing would be completed in the NICU. Researchers then evaluated the testing room environment. A trained nurse was in charge of heel-lancing all neonates in a standardized manner. The heel lancing outcomes were measured at three stages: (1) baseline, T0 (1 min before heel prick), (2) operation, T1 (start to end of heel prick), (3) recovery, and T2(3 min after the operation). One researcher stood to one side, filmed the facial expressions of the newborns, and recorded the heart rate and oxygen saturation values indicated by the ECG monitor during the process. Heart rate and oxygen saturation were recorded every 30 s, and were used to calculate the mean value across the heel prick. Then, two other investigators evaluated the pain scores of the newborns by replaying the continuous physiological and video assessment according to the PIPP-R scale. The PIPP-R was used by two trained nurses who weren’t informed of the purpose of the study to evaluate the pain at each stage of the heel pricks. The score was independently calculated according to the scale, and the final score was taken as the average of the two individuals.

### Interventions

Interventions were provided when the heel lance started, and we continued to provide it until the end of the operation. During the heel pricks, each neonate received corresponding interventions according to their grouping, and neonates in each group were provided the interventions and observed during the tests; then, the data was recorded and analyzed.

#### Control group

Newborns were put in the supine position in the incubator and naked, wearing only diapers. The neonates in the control group were treated the same as the experimental groups provided with routine care. According to the nursing routine, we evaluated the hospital environment before the operation and maintained a quiet environment for all neonates. If newborns were crying, the nurse will comfort the newborn by gentle touch to alleviate procedural pain.

#### NNS group

Each newborn was given a standard solid, soft latex pacifier 1 min before heel prick. The researchers gently supported the pacifier throughout, keeping it in the newborn’s mouth until the end of the test.

#### Maternal voice (MV) group

The newborns in the MV group were provided with a recording of their mother’s voice when the procedure started. The volume inside and outside of the incubator was measured before the procedure to avoid interference by environmental noise, and the recording pen playing the mother’s voice was placed about 15 cm away from the head of the newborn. The volume was measured with a decibel meter, controlled at a range of 50 to 55 dB, according to recommendations [27], and it lasted approximately 10 min. We provided pre-prepared reference recordings, including the song “Little Star,” fetal education stories, and standard script that parents said freely to their child. The recording refers to a singing, a story, or a mother’s voice talking soothingly to her baby.

#### Combined intervention group (NNS + MV)

Both the pacifier and the mother’s voice were implemented at the same time until the end of the heel-prick test.

### Measurements

Demographic data, including gender, gestational age, diagnosis, days of life, weight, and Apgar score, were recorded for the neonates.

The PIPP-R revised by Stevens [28] is used to evaluate the pain of heel blood collection in newborns over 28 weeks old. The PIPP-R is a multidimensional scale including two physiological items (heart rate and oxygen saturation), three behavioral items (frown, eye squeeze, and nasolabial sulcus), and behavioral status and gestational age. Each item is numerically scored on a 4-point scale (0, 1, 2, 3), reflecting increasing changes in each variable from baseline values. Caregivers can rate items from 0 (no pain) to 21 (severe pain). The total score of the scale was the sum of 7 items, with a score of > 12 as severe pain, > 6 as moderate pain, and ≤ 6 indicates that the newborn feels very little pain.

Heart rate and oxygen saturation were recorded by ECG (Dash 1800, GE Medical Systems). Facial expressions were recorded with a camera (Canon PowerShot SX610). Data were recorded in a database (Excel 2007) by a nurse unaware of the study’s aims. The parameters collected included sex, gestational age, days of age, heart rate, oxygen saturation, and PIPP-R scores.

### Ethical considerations

The Institutional Review Board of behavioral and nursing research in the School of Nursing of Central South University approved this study (No. 2017035). The researcher explained the purpose, content, risks, and benefits of this study to the parents of the enrolled neonates, and informed consent was obtained. The American Academy of Pediatrics and the Canadian Pediatric Society recommend sucrose for this painful procedure, but in some developing countries, most infants were not provided any analgesic intervention during painful procedures. The use rate of sucrose tended to be extremely low to prevent pain associated with routine minor procedures, such as heel prick in Chinese neonates. Thus, we did not provide sucrose for this procedure in the study.

### Data analysis

Data were analyzed using Statistical Package for Social Sciences (SPSS) version 21.0. The measurement data follow a normal distribution and are described by means and standard deviations. The counting data were described by frequency and percentage. To analyze the pain scores of the different groups, an analysis of variance was fitted. The significance level was set at 0.05.

## Results

### Participant demographics

A total of 141 newborns were enrolled in the study, with 35 placed in the NNS group, 35 placed in the MV group, 34 placed in the NNS + MV group, and 37 placed in the control group. The neonates ranged from 30 to 41 weeks of gestational age and included 76 males (53.9%) and 65 females (46.1%). There were no significant differences (*P* > 0.05) seen in the comparisons of the general information between groups. Thus, the homogeneity of the four groups was demonstrated (Table 1).

**Table 1:** Descriptive characteristics of participants

Variables	NNS group	MV group	NNS + MV	Control group	*P* value of ${\chi }^{2}/F$
Gender					0.683
Male	26 (61.9)	30 (66.7)	25 (54.3)	28 (59.6)	
Female	16 (38.1)	15 (33.3)	21 (45.7)	19 (40.4)	
Mode of delivery					0.411
Vaginal delivery	21 (50.0)	20 (44.4)	17 (37.0)	16 (34.0)	
Cesarean section	21 (50.0)	24 (55.6)	29 (63.0)	31 (66.0)	
Gestation age (wk)	37.57 ± 2.90	36.81 ± 3.22	36.81 ± 2.97	36.38 ± 3.59	0.376
Apgar score (1 min)	9.31 ± 0.78	9.13 ± 0.86	9.30 ± 0.72	9.28 ± 0.85	0.703
Apgar score (5 min)	9.67 ± 0.61	9.58 ± 0.75	9.70 ± 0.55	9.66 ± 0.73	0.855
Birth weight (kg)	2.89 ± 0.66	2.75 ± 0.79	2.73 ± 0.71	2.58 ± 0.90	0.295
Age of birth (day)	2.52 ± 1.83	2.62 ± 1.51	3.28 ± 2.24	2.90 ± 1.94	0.178
Frequency of heel prick after admission	6.24 ± 4.65	6.98 ± 4.35	6.54 ± 5.34	7.40 ± 5.93	0.666

### The effect of interventions on heel prick pain

#### Heart rate

In this study, before the heel lancing, all neonates at the baseline were quiet and painless; the PIPP-R scores we assessed were all 0 (*P* > 0.05). Therefore, we evaluated changes in the pain scores over time in the operation period. The heart rate values of the four groups during the operation and recovery periods were statistically significant (*P* < 0.05) in Table 2. The Least Significant Difference (LSD) test for comparing the heart rate values of four groups showed that the heart rate values of the control group during the recovery periods were higher than those of the three observation groups and had statistical significance (*P* < 0.05) in Table 3.

**Table 2:** Heart rate of four groups of neonates

Stage	Group	Heart rate	*F*	*P*
Baseline	NNS group	129.64 ± 19.711	0.229	0.876
	MV group	128.33 ± 16.621		
	NNS + MV	131.07 ± 13.940		
	Control group	129.28 ± 13.305		
Operation	NNS group	143.75 ± 19.236	5.098	**0.002**
	MV group	139.29 ± 17.179		
	NNS + MV	140.52 ± 14.023		
	Control group	151.60 ± 16.251		
Recovery	NNS group	135.56 ± 19.970	3.083	**0.029**
	MV group	133.04 ± 17.213		
	NNS + MV	134.24 ± 14.195		
	Control group	142.71 ± 15.763

**Table 3:** LSD test of heart rate in four groups

	(I) Group	(J) Group	Mean Difference (I-J)	Standard Error of Mean	*P*
Heart rate in operation period	Control group	NNS group	7.857	3.549	**0.028**
		MV group	12.314	3.485	**0.001**
		NNS + MV	11.081	3.466	**0.002**
	NNS group	MV group	4.457	3.586	0.215
		NNS + MV	3.224	3.567	0.367
	MV group	NNS + MV	-1.233	3.504	0.725
Heart rate in recovery period	Control group	NNS group	7.154	3.575	**0.047**
		MV group	9.665	3.511	**0.007**
		NNS + MV	8.470	3.492	**0.016**
	NNS group	MV group	2.511	3.612	0.488
		NNS + MV	1.316	3.593	0.715
	MV group	NNS + MV	-1.195	3.530	0.735

#### Blood oxygen saturation (SPO_2_)

The results showed that there was no statistically significant difference in blood oxygen saturation values among the four groups of children during the baseline and recovery periods (*P* > 0.05). The difference in blood oxygen saturation values among the four groups of children during the operation period was statistically significant (*P* < 0.05), as shown in Table 4. The blood oxygen saturation values during the operation period of the three observation groups are higher than those of the control group, and there is statistical significance (*P* < 0.05). The three intervention methods can effectively stabilize the stability of blood oxygen saturation in newborns during the heel blood collection process during the operation period, and there is no statistically significant difference in pairwise comparison between the three intervention methods (*P* > 0.05) in Table 5.

**Table 4:** SPO_2_ of four groups of neonates

Stage	Group	SPO2	*F*	*P*
Baseline	NNS group	94.64 ± 3.574	0.957	0.414
	MV group	94.84 ± 2.645		
	NNS + MV	94.41 ± 2.833		
	Control group	95.45 ± 3.236		
Operation	NNS group	92.86 ± 3.565	4.367	**0.005**
	MV group	92.27 ± 2.630		
	NNS + MV	93.23 ± 3.152		
	Control group	90.87 ± 4.008		
Recovery	NNS group	93.81 ± 3.444	0.860	0.463
	MV group	93.35 ± 2.372		
	NNS + MV	93.79 ± 3.067		
	Control group	92.90 ± 3.537

**Table 5:** LSD test of SPO_2_ in four groups

	(I) Group	(J) Group	Mean Difference (I-J)	Standard Error of Mean	*P*
SPO_2_ in operation period	NNS group	MV group	-1.409	0.705	**0.047**
		NNS + MV	-2.367	0.701	**0.001**
		MV group	0.583	0.725	0.423
		NNS + MV	-0.375	0.721	0.604
	MV group	NNS + MV	-0.958	0.709	0.178

#### PIPP-R score

**Fig. 2:**
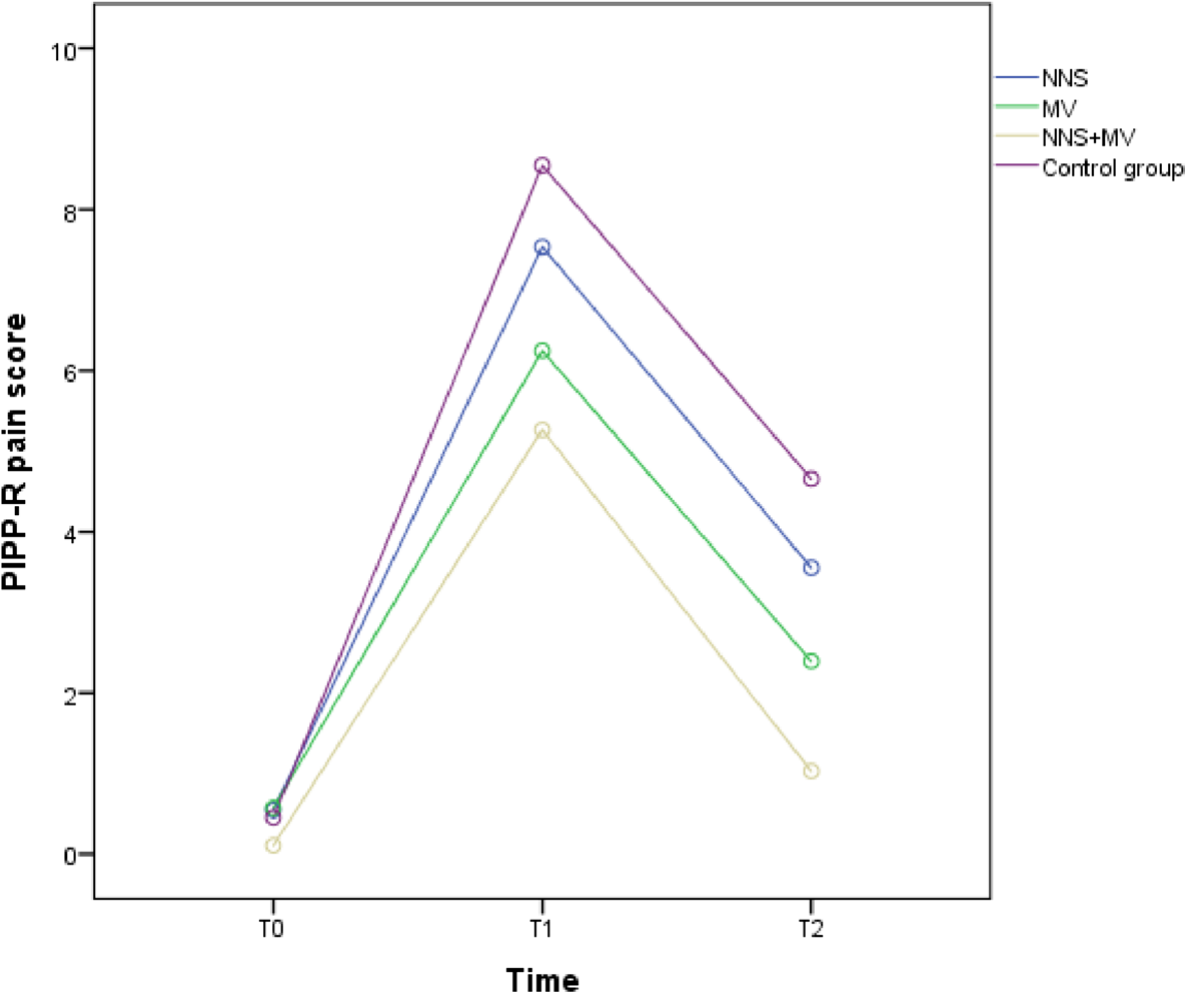
Shows that the pain stimuli experienced by the four groups decreased, but the patterns of changes were different in the procedure period

In the baseline, the PIPP-R score had no difference among the four groups; all the newborns were in a painless state. During the operation, the pain score increased rapidly, and the difference in PIPP-R scores among the four groups of newborns was statistically significant (*P* < 0.05), as shown in Table 6. The changes in PIPP-R score measures in the intervention groups (MV, NNS, and MV + NNS groups) were significant during the operation and recovery period (*P* < 0.05). By further comparing the differences between the three intervention groups, the results showed that the MV + NNS group had a better effect on reducing pain than the other two groups (*P* < 0.05) in Table 7. The MV group had a lower pain score than the NNS group during the recovery period (*P* = 0.004). Combined intervention of maternal voice and non-nutritive sucking could be more effective than any single intervention across repeated procedural pain. The mean PIPP-R score of four groups of children is shown in Fig. 2. The pain score of the control group showed the more significant fluctuation before and after heel prick, compared to the other three groups.

**Table 6:** PIPP-R score of four groups of neonates

Stage	Group	PIPP-R score	*F*	*P*
Baseline	NNS group	0.45 ± 0.149	1.602	0.191
	MV group	0.49 ± 0.144		
	NNS + MV	0.13 ± 0.080		
	Control group	0.49 ± 0.166		
Operation	NNS group	7.79 ± 2.053	20.559	0.000
	MV group	6.36 ± 1.481		
	NNS + MV	5.80 ± 1.827		
	Control group	8.84 ± 2.676		
Recovery	NNS group	3.37 ± 1.902	30.584	0.000
	MV group	2.27 ± 1.188		
	NNS + MV	1.24 ± 1.090		
	Control group	4.41 ± 2.262

**Table 7:** LSD test of PIPP-R score in four groups

	(I) Group	(J) Group	Mean Difference (I-J)	Standard Error of Mean	*P*
PIPP-R score in operation period	NNS group	MV group	2.481	0.430	0.000
		NNS + MV	3.033	0.428	0.000
		MV group	1.438	0.443	0.001
		NNS + MV	1.989	0.440	0.000
	MV group	NNS + MV	0.551	0.432	0.204
PIPP-R score in recovery period	Control group	NNS group	1.038	0.358	0.004
		MV group	2.137	0.351	0.000
		NNS + MV	3.172	0.349	0.000
	NNS group	MV group	1.099	0.361	0.003
		NNS + MV	2.134	0.359	0.000
	MV group	NNS + MV	1.035	0.353	0.004

## Discussion

In this study, we evaluated the effectiveness of the interventions on repeated heel prick pain and compared whether there were differences between the groups and with time. Between-group comparisons revealed that both NNS and the mother’s voice alleviated repeated procedural pain in neonates. Regarding the PIPP-R scores, neonates in the intervention groups showed significantly lower values than newborns in the control group. Additionally, combined NNS and maternal voice stimulation were better for relieving heel prick pain.

In terms of physiological status, the three intervention methods can effectively maintain the stability of vital signs during the heel blood collection process. This study found that all three intervention methods can effectively reduce the fluctuation of heart rate in children during heel blood collection and effectively maintain the stability of blood oxygen saturation in newborns during heel blood collection. This is consistent with the results of many studies on improving the physiological status of newborns when non-nutritive sucking and the mother’s voice are used alone [29–31]. A randomized clinical trial indicated that the non-nutritive sucking applied during heel puncture resulted in effective pain management in newborns and the relationship between heart rate variability and the severity of pain was confirmed [32].

Although a previous study showed that the mother’s voice stimulation improved the physical stability and feeding ability of neonates, the effect of the mother’s voice, and the combined effect of the mother’s voice stimulation and NNS on repeated neonatal pain, had not been examined [21]. Our results are consistent with other studies that demonstrated that the mother’s voice significantly reduces procedural pain among newborns [33, 34]. The maternal voice was recommended as a helpful nursing intervention to reduce pain, decrease heart rate, and increase oxygen saturation in neonates during painful procedures in the NICU [35]. It was assumed that the mother’s voice could modulate newborns’ pain indicators by releasing endogenous oxytocin during vocal contact, which is a promising protective mechanism during early painful interventions in at-risk populations [36]. Therefore, our finding that both mother’s voice stimulation and NNS have a combined effect on repeated heel-stick-induced pain is important.

The most likely explanation for the results is that both NNS and auditory stimulation with mothers’ voices can stabilize infants’ physiologic reactions, probably by reducing their stress reactions [37]. An earlier report on infants of the same age described a combination of oral sucrose and holding as having a stronger effect than oral sucrose alone, and it seems that multiple sensory stimuli work via competition between non-pain stimuli and pain stimuli [38]. Based on the gate-control theory of competition between stimuli for brain perception, we concluded that the mother’s voice and NNS combined may have a greater effect on neonatal pain than single interventions [39]. There is evidence that live music (a lullaby sung by a female) also has positive consequences on long-term outcomes, including the length of hospitalization, weight gain, and NNS [40, 41]. Our study showed that combined NNS and MV stimulation could indeed relieve the pain induced by heel lancing better than their individual use, which is consistent with previous hypothesis. Existing evidence showed that when all elements of sensorial stimulation (tactile, gustatory, auditory and visual) were used, it was more effective than the use of oral sucrose [42]. Although the guidelines of the American Academy of Pediatrics showed that sucrose was safe and effective for reducing procedural pain, the modes of action, optimal dose and long-term effects have not been determined yet [43]. Since oral sucrose use was not common in China, different interventional practices could provide valuable and practical references for Chinese neonates.

Overall, the pain scores in our study are lower than Chirico’s [44] report. They measured the outcome for 10 min when the heel lance procedure was performed. And the length of observation time may affect the average pain score. Newborns, particularly premature babies, are exposed to various kinds of pain and stress in the NICU. The results of our study point to the importance of pain management in neonates with repeated painful procedures. Also, this study found that the maternal sound could be 15 cm away from the newborn’s head in the incubator, and the volume range was 50-55 dB. The mother's voice, including singing and speaking, has a relieving and soothing effect on the pain response of newborns. The difference between the effects of speaking or lullabies is not statistically significant in our study, but the sample size compared in this study is relatively small, and larger sample sizes can be adopted for future research.

A limitation of this study was that blinding could not be performed. In this research, video recordings were used to assess pain, and both the NNS and maternal sound stimulation groups could be easily recognized from the recordings; therefore, we could not prevent possible researcher bias. Besides, healthcare providers could have made the interventions available during non-study heel pricks, which may have affected the results of this study. Furthermore, the sample size was small, and a larger sample size and multicenter randomized controlled trials should be used in the future. Finally, the data were collected several years ago; further research is still needed to compare and analyze the changes between different time periods and regions, in the context of the medical environment that may change under the impact of the epidemic.

Our findings demonstrate that NNS and repeated maternal sounds can be easily incorporated into routine care practices and administered to reduce pain in the NICU setting effectively. These intervention strategies are generally nurse-driven; the use of recorded maternal voices may provide a valuable supplement because it may not be feasible for the mothers to remain at their infant’s bedside all day. Although considering the extensive landscape of pain intervention research over the past decade, encompassing multiple systematic reviews and meta-analyses affirming the benefits of oral sucrose, it was rarely used for neonatal analgesia in the NICU in China. Therefore, this study aimed to explore a simpler and more easily adopted non-pharmacological analgesic intervention more suitable for clinical use in China. NNS and playing recordings of the mother’s voice are recommended for pain management during heel lancing. The effectiveness of NNS and the mother’s voice needs to be further investigated during other kinds of painful procedures and observed for a longer time. Neonates who receive minor but repeated painful stimuli may become irritated during non-painful procedures, and the interventions may help to prevent this.

## Conclusions

This study was conducted to identify the effects of NNS and the mother’s voice on neonate pain scores during heel lancing. Our results provide preliminary evidence that both NNS and the mother’s voice may positively influence the stability of the physiological state and pain relief in neonates during heel prick. Developments derived from this study will enable us to improve NICU care in the future.

## Data Availability

The data sets and materials used and/or analyzed during the study are available from the corresponding author upon reasonable request.

## Abbreviations

NNS: Non-nutritional Sucking
MV: Maternal Voice
NICU: Neonatal Intensive Care Unit
PIPP-R: Preterm Infant Pain Profile-Revised Scale

## References

[1] Maxwell LG, Fraga MV, Malavolta CP (2019). Assessment of pain in the newborn: An update. Clin Perinatol.

[2] Olsson E, Ahl H, Bengtsson K (2021). The use and reporting of neonatal pain scales: a systematic review of randomized trials. Pain.

[3] McPherson C, Miller SP, El-Dib M (2020). The influence of pain, agitation, and their management on the immature brain. Pediatr Res.

[4] Hatfield LA, Murphy N, Karp K (2019). A systematic review of behavioral and environmental interventions for procedural pain management in preterm infants. J Pediatr Nurs.

[5] Cheong JLY, Burnett AC, Treyvaud K (2020). Early environment and long-term outcomes of preterm infants. J Neural Transm (Vienna).

[6] Luo F, Zhu H, Mei L (2023). Evaluation of procedural pain for neonates in a neonatal intensive care unit: a single-centre study. BMJ Paediatr Open.

[7] Li XY, Lee S, Yu HF (2017). Breaking down barriers: enabling care-by-parent in neonatal intensive care units in China. World J Pediatr.

[8] Wang T, Hua Y, Tu H-X, Qiu R, Zhang Q, Yao W-Y (2016). A multicenter study on pediatric nurses' knowledge and attitudes regarding pain management in central china. Chin J Nurs.

[9] Grunau RE (2013). Neonatal pain in very preterm infants: long-term effects on brain, neurodevelopment and pain reactivity. Rambam Maimonides Med J.

[10] Walker SM (2014). Neonatal pain. Paediatr Anaesth.

[11] Harrison D, Sampson M, Reszel J (2014). Too many crying babies: a systematic review of pain management practices during immunizations on YouTube. BMC Pediatr.

[12] Boggini T, Pozzoli S, Schiavolin P (2021). Cumulative procedural pain and brain development in very preterm infants: A systematic review of clinical and preclinical studies. Neurosci Biobehav Rev.

[13] McGinnis K, Murray E, Cherven B (2016). Effect of vibration on pain response to heel lance: A pilot randomized control trial. Adv Neonatal Care.

[14] Committee on Fetus and Newborn and Section on Anesthesiology and Pain Medicine (2016). Prevention and management of procedural pain in the neonate: an update. Pediatrics.

[15] Smith HAB, Besunder JB, Betters KA (2022). 2022 society of critical care medicine clinical practice guidelines on prevention and management of pain, agitation, neuromuscular blockade, and delirium in critically ill pediatric patients with consideration of the icu environment and early mobility. Pediatr Crit Care Med.

[16] Queirós I, Moreira T, Pissarra R, Soares H, Guimarães H (2023). Non-pharmacological management of neonatal pain: a systematic review. Minerva Pediatr (Torino).

[17] Lu M (2014). A systematic review of non-pharmacologic management of heel-stick pain in preterm neonates. Neonatal Netw.

[18] Li XY, Lee S, Yu HF (2017). Breaking down barriers: enabling care-by-parent in neonatal intensive care units in China. World J Pediatr.

[19] Campbell-Yeo M, Fernandes A, Johnston C (2011). Procedural pain management for neonates using nonpharmacological strategies: part 2: mother-driven interventions. Adv Neonatal Care.

[20] Sharma H, Ruikar M (2022). Kangaroo mother care (KMC) for procedural pain in infants: A meta-analysis from the current evidence of randomized control trials and cross-over trials. J Family Med Prim Care.

[21] Shellhaas RA, Burns JW, Barks JDE (2019). Maternal Voice and Infant Sleep in the Neonatal Intensive Care Unit [published correction appears in Pediatrics. 2020 May; 145(5). Pediatrics.

[22] Williamson S, McGrath JM (2019). What are the effects of the maternal voice on preterm infants in the NICU?. Adv Neonatal Care.

[23] Jin L, Zhang J, Yang X (2023). Maternal voice reduces procedural pain in neonates: A meta-analysis of randomized controlled trials. Medicine (Baltimore).

[24] Picciolini O, Porro M, Meazza A, Gianni L, Rivoli C (2014). Early exposure to maternal voice: Effects on preterm infants development. Early Hum Dev.

[25] Liaw JJ, Yang L, Lee CM (2013). Effects of combined use of non-nutritive sucking, oral sucrose, and facilitated tucking on infant behavioural states across heel-stick procedures: A prospective, randomised controlled trial. Int J Nurs Stud.

[26] Barandouzi ZA, Keshavarz M, Montazeri A (2020). Comparison of the analgesic effect of oral sucrose and/or music in preterm neonates: a double-blind randomized clinical trial. Complement Ther Med.

[27] Graven SN (2000). Sound and the developing infant in the NICU: conclusions and recommendations for care. J Perinatol.

[28] Stevens BJ, Gibbins S, Yamada J (2014). The premature infant pain profile-revised (PIPP-R): initial validation and feasibility. Clin J Pain.

[29] García-Valdivieso I, Yáñez-Araque B, Moncunill-Martínez E (2023). Effect of non-pharmacological methods in the reduction of neonatal pain: systematic review and meta-analysis. Int J Environ Res Public Health.

[30] Yu WC, Chiang MC, Lin KC (2022). Effects of maternal voice on pain and mother-Infant bonding in premature infants in Taiwan: A randomized controlled trial. J Pediatr Nurs.

[31] Queirós I, Moreira T, Pissarra R (2023). Non-pharmacological management of neonatal pain: a systematic review. Minerva Pediatr (Torino).

[32] Napiórkowska-Orkisz M, Gutysz-Wojnicka A, Tanajewska M (2022). Evaluation of methods to minimize pain in newborns during capillary blood sampling for screening: a randomized clinical trial. Int J Environ Res Public Health.

[33] Azarmnejad E, Sarhangi F, Javadi M, Rejeh N (2015). The effect of mother's voice on arterial blood sampling induced pain in neonates hospitalized in neonate intensive care unit. Glob J Health Sci.

[34] Ardesi M, Dioni E (2017). Randomised study showed that recorded maternal voices reduced pain in preterm infants undergoing heel lance procedures in a neonatal intensive care unit. Acta Paediatr.

[35] Erdoğan Ç, Turan T, Pınar B (2020). The effect of maternal voice for procedural pain in paediatric intensive care unit: A randomised controlled trial. Intensive Crit Care Nurs.

[36] Filippa M, Monaci MG, Spagnuolo C, Serravalle P, Daniele R, Grandjean D (2021). Maternal speech decreases pain scores and increases oxytocin levels in preterm infants during painful procedures. Sci Rep.

[37] Williamson S, Mcgrath JM (2019). What are the effects of the maternal voice on preterm infants in the NICU?. Adv Neonatal Care.

[38] Cignacco E, Sellam G, Stoffel L (2012). Oral sucrose and "facilitated tucking" for repeated pain relief in preterms: A randomized controlled trial. Pediatrics.

[39] Mendell LM (2014). Constructing and deconstructing the gate theory of pain. Pain.

[40] Polkki T, Korhonen A (2012). The effectiveness of music on pain among preterm infants in the neonatal intensive care unit: a systematic review. JBI Libr Syst Rev.

[41] Bellieni CV, Tei M, Coccina F (2012). Sensorial saturation for infants’ pain. J Matern Fetal Neonatal Med.

[42] Lim Y, Godambe S (2017). Prevention and management of procedural pain in the neonate: an update, American Academy of Pediatrics, 2016. Arch Dis Child Educ Pract Ed.

[43] Chorna O, Filippa M, De Almeida JS (2019). Neuroprocessing mechanisms of music during fetal and neonatal development: a role in neuroplasticity and neurodevelopment. Neural Plast.

[44] Chirico G, Cabano R, Villa G (2017). Randomised study showed that recorded maternal voices reduced pain in preterm infants undergoing heel lance procedures in a neonatal intensive care unit. Acta Paediatr.

